# Uso Concomitante de Ranolazina e Trimetazidina em Pacientes com Angina Refratária: Uma Experiência Inicial

**DOI:** 10.36660/abc.20210711

**Published:** 2022-07-28

**Authors:** Luciana Oliveira Cascaes Dourado, Cristian Paul Delgado Moreno, Sarah Fagundes Grobe, Luis Henrique Wolff Gowdak, Luiz Antonio Machado Cesar

**Affiliations:** 1 Hospital das Clínicas Faculdade de Medicina Universidade de São Paulo São Paulo SP Brasil Instituto do Coração do Hospital das Clínicas da Faculdade de Medicina da Universidade de São Paulo, São Paulo, SP – Brasil

**Keywords:** Doença Arterial Coronariana, Angina Refratária, Tratamento Farmacológico, Ranolazina/uso terapêutico, Trimetazidina/uso terapêutico

## Introdução

A angina refratária (AR), uma condição extremamente debilitante, requer tratamento médico especializado com ajustes terapêuticos muitas vezes complexos na tentativa de melhorar ao máximo os sintomas e a qualidade de vida.^1^

O tratamento médico geralmente compreende uma combinação de medicamentos antianginosos. Dentre eles, a trimetazidina (T) e a ranolazina (R) são uma terapia complementar devido ao seu perfil de eficácia e segurança no tratamento de pacientes com AR.^1 - 3^ No entanto, na recente “abordagem diamante”,^4^ que descreve combinações preferenciais de diferentes classes de medicamentos antianginosos para o tratamento de pacientes com angina, o uso concomitante de T e R não é considerado uma estratégia útil devido ao seu mecanismo de ação relacionada.^4^ Embora nenhuma interação conhecida entre ambas as drogas tenha sido descrita,^5^ não há dados sobre a eficácia e segurança do uso de R em pacientes que já estão usando T. Portanto, objetivamos avaliar o efeito do uso concomitante de R e T em pacientes com AR.

## Métodos

Analisamos retrospectivamente os prontuários clínicos de pacientes acompanhados em um ambulatório especializado de um hospital universitário terciário com diagnóstico de AR, definida como angina incapacitante com pelo menos 3 meses causada por insuficiência coronariana em quadro de doença arterial coronariana,^6 , 7^ confirmada por angiografia e em pacientes não elegíveis para revascularização miocárdica.^8^ Uma amostragem de conveniência de pacientes que estavam sintomáticos após 3 meses de uso de pelo menos 3 medicamentos antianginosos (incluindo T) foi elegível para receber R. Esta análise fez parte de um estudo aprovado pelo comitê de ética em pesquisa (CAAE:24308213.7.0000.0068). As investigações seguiram a Declaração de Helsinque. Todos os pacientes forneceram consentimento informado por escrito.

Os pacientes foram reavaliados mensalmente durante 3 meses (visita inicial: V_1_; última visita: V_4_) quanto aos seus sintomas de acordo com a classificação da Canadian Cardiovascular Society (CCS). ECG de repouso e exames laboratoriais foram realizados em V_1_ e V_4_. A critério do médico, era possível adicionar R à terapia de base^9^ se a) QT_c_ < 500 ms; b) taxa de filtração glomerular > 30 mL.kg^-1^.min^-1^; e c) ausência de disfunção hepática grave. Foi adotada uma dose padrão de 500 mg duas vezes ao dia.

### Análise estatística

A análise estatística foi realizada usando IBM SPSS, versão 20. As variáveis apresentaram distribuição normal de acordo com o teste de normalidade de Shapiro-Wilk. Portanto, as variáveis contínuas foram expressas como média ± desvio padrão (DP) e as variáveis categóricas foram expressas como números absolutos. Para comparação entre os pontos temporais, foi utilizado o teste t de Student pareado ou o teste de postos sinalizados de Wilcoxon, conforme apropriado. A significância estatística foi estabelecida como valor p < 0,05.

## Resultados

Este relato inicial avaliou 10 pacientes (7 homens), 61 ± 7 anos, acompanhados por angina limitante com CCS 3 (n = 3) ou 4 (n = 7), entre 2019 e 2020. A Tabela 1 mostra suas características basais. No ECG de repouso basal, a frequência cardíaca era de 64 ± 7 bpm e o QT_c_ era de 414 ± 16 ms.

**Tabela 1:** Dados clínicos, eletrocardiográficos e laboratoriais de V1 a V4

Variável	V_1_	V_4_	p
**Fatores de risco cardiovascular**			
Hipertensão (n)	6		
Diabetes mellitus (n)	5		
Hiperlipidemia (n)	10		
Tabagismo (anterior ou atual) (n)	7		
Obesidade (n)	3		
Histórico familiar de DAC (n)	3		
**Histórico médico**			
Tempo de diagnóstico de DAC, anos (média ± DP)	8,7±6,0		
Infarto agudo do miocárdio (n)	8		
Intervenção coronária percutânea (n)	9		
CRM (n)	4		
**Padrão obstrutivo e função do VE**			
FEVE (ecocardiografia), (média ± DP)	0,56±0,07		
Doença de um vaso (n)	2		
Doença de dois vasos (n)	3		
Doença de três vasos (n)	5		
**Medicamentos**			
Aspirina (n)	10		
Clopidogrel (n)	4		
Estatina (n)	10		
% dosagem máxima (média ± DP)	100		
Betabloqueadores (n)	9		
% dosagem máxima (média ± DP)	100		
Bloqueadores dos canais de cálcio (n)	10		
% dosagem máxima (média ± DP)	80±26		
Nitratos de ação prolongada (n)	10		
% dosagem máxima (média ± DP)	90±23		
Trimetazidina (n)	10		
Ivabradina (n)	2		
Inibidores da enzima conversora de angiotensina (n)	1		
Bloqueadores dos receptores de angiotensina (n)	4		
% dosagem máxima (média ± DP)	100		
Diuréticos, tiazidas (n)	4		
Medicamentos antidiabéticos orais (n)	4		
Insulina (n)	2		
**Dados clínicos**			
Pressão arterial sistólica, mmHg (média ± DP)	122±17	118±17	0,5
Pressão arterial diastólica, mmHg (média ± DP)	75±5	71±9	0,1
Frequência cardíaca, bpm (média ± DP)	65±5	62±10	0,7
**ECG**			
Frequência cardíaca, bpm (média ± DP)	64±7	61±9	0,39
QT_c,_ ms (média ± DP)	414±16	417±19	0,44
**Laboratório**			
Hemoglobina, g/dL (média ± DP)	13,4±1,7	13,9±0,9	0,2
Creatinina, mg/dL (média ± DP)	1,06±0,20	1,08±0,10	0,6
HbA1_C_, % (média ± DP)	7,1±2,5	6,7±1,3	0,4
LDL-colesterol, mg/dL (média ± DP)	91±43	96±33	0,9
HDL-colesterol, mg/dL (média ± DP)	42±9	43±11	0,7
Triglicerídeos, mg/dL (média ± DP)	118±27	118±32	0,9
ALT, mg/dL (média ± DP)	26±8	28±8	0,8
AST, mg/dL (média ± DP)	20±4	20±6	0,6
Sódio, mmol/L (média ± DP)	139±3	141±2	0,07
Potássio, mmol/L (média ± DP)	4,7±0,5	4,8±0,4	0,8

*ALT: alanina aminotransferase; AST: aspartato aminotransferase; CRM: cirurgia de revascularização do miocárdio; DAC: doença arterial coronariana; DP: desvio padrão; ECG: eletrocardiograma; FEVE: fração de ejeção do ventrículo esquerdo; Hb: hemoglobina; HDL: lipoproteína de alta densidade; LDL: lipoproteína de baixa densidade; V: visita; VE: ventrículo esquerdo.*

Com a exceção de 1 paciente, todos compareceram às visitas agendadas. Em V_2_, 4 dos 10 pacientes apresentaram hipotensão sintomática levando a uma mudança no tratamento anti-hipertensivo: os anti-hipertensivos tiveram que ser interrompidos em 2 pacientes em uso de anlodipino ou hidroclorotiazida ou reduzidos em um paciente em uso de losartana. Na V_4_, apenas 1 paciente faltou à consulta e, embora tenha sido contatado por telefone, confirmando que estava clinicamente estável, essa informação não foi incluída na análise.

A Tabela 2 mostra a CCS dos pacientes individualmente em cada visita. Observamos melhora significativa na CCS de V_1_ para V_4_ (Z= −2,07; p = 0,038). Dois pacientes melhoraram 2 classes de CCS e 3 pacientes melhoraram 1 classe. No entanto, 4 pacientes não apresentaram melhora.

**Tabela 2:** CCS de linha de base e de V4 dos pacientes individualmente

Paciente	CCS		p^*^
	V_1_	V_4_	0,038
1	3	2	
2	3	1	
3	4	4	
4	4	3	
5	4	4	
6	3	3	
7	4	3	
8	4	-	
9	4	2	
10	4	4	

CCS: Canadian Cardiovascular Society; V: visita. * Teste dos postos sinalizados de Wilcoxon

A análise dos ECGs de repouso obtidos em V_4_ não revelou alterações significativas na frequência cardíaca (61 ± 9 bpm) e QT_c_ (417 ± 19 ms) em relação à linha de base (valores p de 0,39 e 0,44, respectivamente).

## Discussão

Até onde sabemos, este é o primeiro relato do uso combinado de R (inibidor da corrente de Na^+^ tardia) com T (inibidor parcial da oxidação de ácidos graxos livres) para otimizar o tratamento médico em pacientes com AR. Nesta experiência inicial, a combinação foi segura e bem tolerada e resultou em uma melhora da CCS.

Devido à persistência da angina limitante apesar da associação de pelo menos três medicamentos antianginosos, incluindo T, a introdução de R na tentativa de melhor controlar os sintomas foi cuidadosamente monitorada. O único evento adverso observado após R foi hipotensão sintomática, embora R seja presumivelmente livre de qualquer efeito hemodinâmico significativo. A possível explicação para a hipotensão vem das muitas interações medicamentosas atribuídas a R que pode, por exemplo, diminuir a taxa de excreção de losartana, o que poderia resultar em um nível sérico mais elevado.^5^ Da mesma forma, a concentração sérica de levanlodipino pode ser aumentada quando combinada com R. No ensaio CARISA,^9^ a incidência de hipotensão foi em torno de 1%, muito inferior à observada em nosso estudo. A única interação relatada entre R e hidroclorotiazida é risco ou gravidade maior de prolongamento do intervalo QT_c_.

Três meses após a introdução do R, observamos melhora significativa na CCS e ausência de prolongamento do intervalo QT_c_ ou anormalidades laboratoriais, sugerindo que o uso concomitante de medicamentos antianginosos atuando tanto no nível celular quanto cardíaco é seguro e oferece alívio adicional da angina. A mensagem central dessa experiência inicial é que, ao enfrentar um desafio médico, o clínico deve ser cautelosamente audacioso. Acreditamos que novas estratégias, desde que seguras e baseadas em um raciocínio lógico, devem ser consideradas, com o objetivo de levar esperança aos pacientes ditos “sem opção” .

### Limitações

Os achados do presente estudo devem ser vistos à luz de algumas limitações. Nosso estudo é uma análise retrospectiva dos dados de uma amostra pequena, de um ensaio aberto, avaliando o desfecho subjetivo de sintomas de angina em pacientes com AR acompanhados por 3 meses. Portanto, embora nossas conclusões não sejam definitivas, elas geram hipóteses para ensaios futuros.

## Conclusões

O uso concomitante de R e T em pacientes com AR, durante 3 meses, melhorou a CCS e foi seguro, sem evidência de prolongamento do QT_c_ ou anormalidades laboratoriais.
