# Supplementary material for: Uso Concomitante de Ranolazina e Trimetazidina em Pacientes com Angina Refratária: Uma Experiência Inicial
Source: Arq Bras Cardiol. 2022 Jul 28;119(4):619–21. [Article in Portuguese] doi: 10.36660/abc.20210711 (PMC9563874; doi:10.36660/abc.20210711)
Supplement: Translation

# Translation: Concomitant Use of Ranolazine and Trimetazidine in Patients with Refractory Angina: An Initial Experience

**Authors:** Luciana Oliveira Cascaes Dourado, Cristian Paul Delgado Moreno, Sarah Fagundes Grobe, Luis Henrique Wolff Gowdak, Luiz Antonio Machado Cesar

**Affiliations:** 1 Brazil Instituto do Coração do Hospital das Clínicas da Faculdade de Medicina da Universidade de São Paulo, São Paulo, SP – Brazil

**Keywords:** Coronary Artery Disease, Refractory Angina, Drug Therapy, Ranolazine/therapeutic use, Trimetazidine/therapeutic use

## Introduction

Refractory angina (RA), an extremely debilitating condition, requires specialized medical treatment with often complex therapeutic adjustments in an attempt to improve symptoms and quality of life as much as possible.^1^

Medical treatment usually comprises a combination of antianginal drugs (AAD). Among them, trimetazidine (T) and ranolazine (R) are an add-on therapy due to their efficacy and safety profile in the treatment of patients with RA.^1 - 3^ However, in the recent “diamond approach”,^4^ depicting preferred combinations of different classes of AAD for the treatment of patients with angina, does not consider the concomitant use of T and R a useful strategy due to their related mechanism of action.^4^ Although no known interaction between both drugs has been described,^5^ there is no data on the efficacy and safety of the use of R in patients already on T. Therefore, we aimed to evaluate the effect of the concomitant use of R and T in patients with RA.

## Methods

We retrospectively analyzed the clinical records of patients followed in a specialized, outpatient clinic of a tertiary university hospital with diagnosis of RA, defined as disabling angina for at least 3 months caused by coronary insufficiency in the setting of coronary artery disease,^6 , 7^ confirmed by angiography and in patients not eligible for myocardial revascularization.^8^ A convenience sampling of patients who were symptomatic after 3 months of at least 3 AAD (including T) were eligible to receive R. This analysis was part of a study approved by the research ethics committee (CAAE:24308213.7.0000.0068). Investigations followed the Declaration of Helsinki. All patients provided written informed consent.

Patients were reassessed monthly for 3 months (baseline visit: V_1_; last visit: V_4_) regarding their symptoms according to the Canadian Cardiovascular Society (CCS). Resting ECG and laboratory tests were performed on V_1_and V_4_. At the physician’s discretion, R could be added to the background therapy^9^ if a) QT_c_< 500 ms; b) glomerular filtration rate > 30 mL.kg^-1^.min^-1^; and c) absence of severe hepatic dysfunction. A standard dose of 500 mg twice daily was adopted.

### Statistical analysis

Statistical analysis was performed using IBM SPSS, version 20. Variables presented a normal distribution according to Shapiro-Wilk normality test. Therefore, continuous variables were expressed as mean ± standard deviation (SD), and categorical variables were expressed as absolute numbers. For comparison between time points, the paired Student t-test or Wilcoxon signed-rank test were used, as appropriate. Statistical significance was set at a p-value of < 0.05.

## Results

This early report evaluated 10 patients (7 men), 61 ± 7 years old, followed for limiting angina CCS 3 (n = 3) or 4 (n = 7), between 2019 and 2020. Table 1 shows their baseline characteristics. On baseline resting ECG, heart rate was 64 ± 7 bpm, and QT_c_was 414 ± 16 ms.

**Table 1:** Clinical, electrocardiographic, and laboratory data from V1 to V4

Variable	V_1_	V_4_	p
**Cardiovascular risk factors**			
Hypertension (n)	6		
Diabetes mellitus (n)	5		
Hyperlipidemia (n)	10		
Smoking (previous or current) (n)	7		
Obesity (n)	3		
Family history of CAD (n)	3		
**Past medical history**			
CAD diagnostic time, years (mean ± SD)	8.7±6.0		
Acute myocardial infarction (n)	8		
Percutaneous coronary intervention (n)	9		
CABG (n)	4		
**Obstructive pattern and LV function**			
LVEF (echocardiography), (mean ± SD)	0.56±0.07		
One-vessel disease (n)	2		
Two-vessel disease (n)	3		
Three-vessel disease (n)	5		
Medication			
Aspirin (n)	10		
Clopidogrel (n)	4		
Statin (n)	10		
% maximum dosage (mean ± SD)	100		
β-blockers (n)	9		
% maximum dosage (mean ± SD)	100		
Calcium channel blockers (n)	10		
% maximum dosage (mean ± SD)	80±26		
Long-acting nitrates (n)	10		
% maximum dosage (mean ± SD)	90±23		
Trimetazidine (n)	10		
Ivabradine (n)	2		
Angiotensin-converting-enzyme inhibitors (n)	1		
Angiotensin receptor blockers (n)	4		
% maximum dosage (mean ± SD)	100		
Diuretics, thiazides (n)	4		
Oral antidiabetic drugs (n)	4		
Insulin (n)	2		
**Clinical data**			
Systolic blood pressure, mmHg (mean ± SD)	122±17	118±17	0.5
Diastolic blood pressure, mmHg (mean ± SD)	75±5	71±9	0.1
Heart rate, bpm (mean ± SD)	65±5	62±10	0.7
**ECG**			
Heart rate, bpm (mean ± SD)	64±7	61±9	0.39
QT_c_, ms (mean ± SD)	414±16	417±19	0.44
**Laboratory**			
Hemoglobin, g/dL (mean ± SD)	13.4±1.7	13.9±0.9	0.2
Creatinine, mg/dL (mean ± SD)	1.06±0.20	1.08±0.10	0.6
Hb1_C_, % (mean ± SD)	7.1±2.5	6.7±1.3	0.4
LDL-cholesterol, mg/dL (mean ± SD)	91±43	96±33	0.9
HDL-cholesterol, mg/dL (mean ± SD)	42±9	43±11	0.7
Triglycerides, mg/dL (mean ± SD)	118±27	118±32	0.9
ALT, mg/dL (mean ± SD)	26±8	28±8	0.8
AST, mg/dL (mean ± SD)	20±4	20±6	0.6
Sodium, mmol/L (mean ± SD)	139±3	141±2	0.07
Potassium, mmol/L (mean ± SD)	4.7±0.5	4.8±0.4	0.8

*ALT: alanine aminotransferase; AST: aspartate aminotransferase; CABG: coronary artery bypass graft; CAD: coronary artery disease; ECG: electrocardiogram; Hb: hemoglobin; HDL: high-density lipoprotein; LDL: low-density lipoprotein; LV: left ventricular; LVEF: left ventricular ejection fraction; SD: standard deviation; V: visit.*

All but one patient attended the scheduled visits. At V_2_, 4 of the 10 patients presented symptomatic hypotension leading to a change in antihypertensive treatment: antihypertensive drugs had to be either stopped in 2 patients using amlodipine or hydrochlorothiazide or reduced in one patient using losartan. At V_4_, a single patient missed his appointment, and, although he was reached by phone, confirming he was clinically stable, this information was not included in the analysis.

Table 2 shows CCS of patients individually at each visit. We observed significant improvement in CCS from V_1_to V_4_(Z= −2.07; p = 0.038). Two patients improved 2 CCS classes, and 3 patients improved 1 class. However, 4 patients exhibited no improvement.

**Table 2:** Baseline and V4 CCS of patients individually

Patient	CCS		p^*^
	V_1_	V_4_	0.038
1	3	2	
2	3	1	
3	4	4	
4	4	3	
5	4	4	
6	3	3	
7	4	3	
8	4	-	
9	4	2	
10	4	4	

CCS: Canadian Cardiovascular Society; V: visit. * Wilcoxon signed-rank test.

Analysis of the resting ECGs obtained at V_4_disclosed no significant changes in heart rate (61 ± 9 bpm) and QT_c_(417 ± 19 ms) compared to baseline (p-values of 0.39 and 0.44, respectively).

## Discussion

To our knowledge, this is the first report on the combined use of R (a late Na^+^current inhibitor) with T (a partial free fatty acid oxidation inhibitor) to optimize medical treatment in patients with RA. In this early experience, the combination was safe and well tolerated, and it led to an improvement in CCS.

Because of the persistence of limiting angina despite the association of at least three AAD, including T, the introduction of R in an attempt to better control symptoms was carefully monitored. The only observed adverse event after R was symptomatic hypotension, although R is presumably devoid of any significant hemodynamic effect. The possible explanation for hypotension comes from the many drug-to-drug interactions ascribed to R. Ranolazine, for instance, may decrease the excretion rate of losartan which could result in a higher serum level.^5^ Likewise, the serum concentration of levamlodipine can be increased when it is combined with R. In the CARISA trial,^9^ the incidence of hypotension was around 1%, much lower than that observed in our study. The only reported interaction between R and hydrochlorothiazide is an increased risk or severity of QT_c_prolongation.

Three months after the introduction of R, we observed significant improvement in CCS and no QT_c_prolongation or laboratory abnormalities, suggesting that the concomitant use of AAD acting both at the cardiac cell level is safe and offers additional angina relief. The central message of this early experience is that, when facing a medical challenge, the clinician must be cautiously audacious. We believe that new strategies, provided they are safe and based on a logical rationale, must be considered, which the aim of bringing hope to so-called “no-option” patients.

### Limitations

The findings of this study have to be seen in light of some limitations. Our study is a retrospective analysis of the data of a small sample size, of an open trial, evaluating the subjective endpoint of angina symptoms in patients with RA followed during 3 months. Therefore, although our conclusions are not definite, they generate hypotheses for future trials.

## Conclusions

Concomitant use of R and T in patients with RA, during 3 months, improved CCS and was safe, with no evidence of QT_c_prolongation or laboratory abnormalities.
